# Design and implementation of thermal collection networks in 3-D IC structures

**DOI:** 10.1016/j.heliyon.2022.e08719

**Published:** 2022-01-10

**Authors:** Chandrashekhar V. Patil, M.S. Suma

**Affiliations:** a Department of Electronics and Communication Engineering, B.M.S. College of Engineering, Visveswaraya Technological University, Bangalore, 560019, India; b Department of Medical Electronics Engineering, B.M.S. College of Engineering, Visveswaraya Technological University, Bangalore, 560019, India

**Keywords:** Coefficient of thermal efficiency, Monolithic 3-D IC, Thermal collection network, Thermal integrity, Thermal resistance

## Abstract

The empirical affirmation in the electronics industry is that the power of chips per unit area is growing exponentially. The amount of heat generated is equal to the power; hence as power per unit area increases, so does the amount of heat generated within the chip. Thus, it necessary to mitigate the thermal problems of electronic systems. If not addressed or suppressed, thermal problems can lead to various issues including dielectric breakdown, electromigration, material creeping, unwanted chemical reactions, board warpage, drift in performance, and indirect heating. In this study, a dedicated thermal collection network (TCN) in the back end of the line area of an electronic chip was investigated. This network can help in creating a connection using a thermal through Silicon via (TTSV) to pump up the thermal energy to the heat-sink–fan assembly. Pre-empting heat from the sources could manage the thermal issues arising in chips as well as three-dimensional integrated circuit (3-D IC) structures. The finite-element method was the tool used for analysis. 31.62% of heat suction in TCNs of monolithic ICs, 11.36% in TCNs of 3-D IC structures, and 35.34% of heat suction in junctions of TTSVs compared with different approaches without the postulate used here. This procedure is expected to lead to a new path for redesigning electronic chips and 3-D IC structures.

## 1 Introduction

When a chip is designed to meet the static current–resistance (IR) drop, typical power grid and power switches are added. Achieving a robust power grid architecture is difficult, even when module-based decoupling capacitor (decap) cells and module-based control switches are present. To solve the issue, different techniques must be tested. Based on the logic switching operation for a specific period, a hotspot could form because of the massive dynamic current drawn by localized logic areas.

These hotspots can be fixed through decap cells while constraining them to a particular percentage of targeted voltages. However, it has been shown that these hotspots are important attributes in the process of thermal energy accumulation. They help in distributing thermal energy to the integrated wires and substrates of the chip by inducing indirect heat to the chip logic as well as other components. In such a situation, the IR drop targeted by the decap cells improvises on the advanced nodes, and it would be minimal.

According to Singh and Tan [1], a thermal through silicon via (TTSV) is used within three stacked assemblies for reducing the chip's maximum temperature to within the range of 54 °C–62 °C. Nithin *et al.* [2], discussed various techniques for reducing the dynamic voltage drop, which is widely used in the industry. However, padding with decap cells around the clock tree cells, as well as the highly switching input/output buffers, is affected by effective dynamic voltage drop and already has been demonstrated. It can help in reducing the dynamic voltage drop. Metal fill is another method that has been explored in this research field. Augmentation of floating metal straps could be performed by connecting them to power or ground. The power and ground grids become stronger as a result. Consequently, the dynamic voltage drop is minimized. Using computational fluid dynamics methods, Lau and Yue [3] demonstrated a decrease in chip temperature when through silicon vias (TSVs) were used; they also observed a rise in junction temperature when a larger number of TSVs were used in three-dimensional (3-D) stacked systems. Al Qahtani *et al.* [4] performed a hotspot validation and thermal analysis in a case study of an industry-standard architecture. This assisted in the development of the proposed design for thermal collection networks (TCNs). Chai *et al.* [5] investigated the thermal efficiency method in a stacked integrated circuit (IC) system. The thermal conductivity was investigated and compared with different published results. Various packaging schemes available in 3-D IC structures were explored by Yann *et al.* [6] and by Osmolovskyi and Dresden [7]. TSVs were used to demonstrate wafer-level packaging and physical design challenges in 3-D IC packages. The study focused on the electronic design of automation tools that are needed to assist in the design of such packages, including the TCNs connecting to TSVs. The study also showed that the TCNs could be laid right after the power grid before the chip design clock/net routing processes. Using carbon nanotube injection inside the filler material, Patel *et al.* [8], investigated the von Mises stress generated on such a TTSV, which can be reduced by 40 MPa. As a result of this reduction, the reliability of the chip improved. Kaddi *et al.* [9] suggested an active cooling technique within the TTSV with a Peltier element, which could be a better solution for heat removal in 3-D ICs than the passive techniques discussed so far. Agrawal *et al.* [10] conducted a complete case study on the Xylem chip, placing the TTSVs in the stack and measuring the temperature. A case study with multiple memory chips was conducted by Ren *et al.* [11]. They discovered that the optimal stack for lowering the peak temperature was based on the TTSV and power-deficient areas of the chip.

The TTSV is not a new concept; however, the present study is the first to use the proposed TCN concept. The use of a greater number of vertical TTSVs in the areas where power is consumed has been proposed. Although this works well for large network processors/chips, the technique has some disadvantages, including the possibility of wide-area overhead and coefficient of thermal expansion (CTE). With these disadvantages in mind, active devices, such as fluidic solutions and interposer techniques, are considered well-suited for industrial development. Unfortunately, based on available literature reviews, there has been no attempt to create thermal runway paths for rapid heat absorption within a chip or in 3-D IC structures effectively and efficiently. In this study, such a use of devices for thermal management is proposed based on a careful examination of the root cause of heat and regions of heat sources on the chip. In addition, the term “thermal collection network” is introduced in this article. The methodology is to vary the number of TCN grids from zero to three during the investigation. These are dispersed across the three vertical regions of the chip. The number of TTSVs that link to these regions vary from zero to five, and they are connected using different TCN grid arrangements.

In Section 2, the size of the widths of TCNs is discussed. The modeling of TCNs is described in Section 3. The findings are discussed in Section 4. Using computations and finite-element method (FEM) tool readings, the thermal efficiency of the TCN, parameters, and various results are described. In Section 5, the results are summarized and related conclusions are drawn.

## 2 Design and thermal models of TCNs

Bhooshan [12] developed power models based on IR drop as well as electromigration (EM) calculations. Similarly, a calculation method for the width of the thermal collection metal could be developed by sustaining currents (by taking a resistance as the thermal representation) in a given thermal budget. Via resistances are insignificant here because of their short length. Moreover, double via insertions would lower the resistance of vias that are connected in a parallel manner in TCNs, which is a common industry practice. A TCN can be connected to the ground so that the TTSV can be connected to ground paths of current to alleviate the capacitance of the metals.

(1) $$W_{IR}=\frac{min\;\left(I_{chip},\;I_{EM}\right)\;\text{L}}{2\;\partial_{IR}\;V_{DD\;}\;G\;\sum_{n=1}^{\infty }g\left(n\right)}\;$$

(2) $$W_{EM}=max\left\{\frac{min\;\left(I_{chip},\;I_{EM}\right)\;g\left(n\right)}{2\;\left[\sum_{n=1}^{\infty }g\left(n\right)\right]\;J_{EM}\left(n\right)\;}\right\},\;\forall_{n}$$

Here, the number of metal layers used in the core rings is denoted by n, g(n) is the normalized conductivity of the *n*th metal layer, G(n) is the equivalent conductivity of the *n*th metal layer, *L* is the distance between two ${\text{ ​V}}_{\text{DD ​}}$ pad cells, and $J_{EM}\left(n\right)\;\text{i}$ s the electromigration current density in the limit of the n th metal layer. The minimum current ($I_{chip}\;$ or $I_{EM}$) carried to meet the IR drop budget is $\partial_{IR},\text{and}\;{\text{ ​V}}_{\text{DD}}\text{ ​is ​the ​supply ​voltage}$. Moreover, ${\text{W}}_{\text{EM}}$ is the width of the metal, calculated according to the EM, and $W_{IR}$ is the width of the metal calculated according to the IR drop budget.

To prevent charge accumulation, TTSVs can be electrically grounded, as previously mentioned. When connecting TCNs to the chip power or ground supplies (augmentation), the widths can be calculated using Eqs. (1) and (2). In such cases, whichever equation gives a larger value can be considered to give the actual widths of the TCN rings to sustain both IR and EM currents. The scope of this study is the budget of the thermal energy in terms of the resistance models solved for the widths of metals if the TCNs are not connected electrically.

## 3 Modeling setup

The finite-element approach is a mathematical simulation technique specifically designed for fluid flow, heat transfer, and many other physical systems. Using FEM, Ansys Workbench 2.0 software, 2020-R1 [13], was used for quantitative and qualitative predictions on heat transfer flow and several other phenomena using partial differential equations. The steady-state thermal analysis was done using this tool. First, the simulation process involves drawing the geometries of the system. Second, material assignments were followed by constraining boundary conditions. Meshing was the final step in the process, followed by simulation. SolidWorks 2020 [14] was used to build the geometries. The aforementioned software is used to build computer-aided designs in two and three dimensions.

A copper-filled TSV was thermally evaluated by Chien *et al.* [15]. It was considered as a unit cell, with a thickness of T and a pitch of P. The equivalent thermal conductivity of this TSV cell was derived. The thermal conductivities to be calculated include planes along the $k_{xy}$ direction (left to the right wall, i.e., horizontal thermal flow) and $k_{z}$ direction (top to bottom, i.e., vertical thermal flow) in the cross section of the TSV/TTSV. The positive heat flux $Q_{in}$ is applied to one of its side surfaces (left wall), and $-Q_{out}$, a negative flux, is applied to the opposite side of the surface (right wall). Applying Fourier's law of thermal conduction, the equivalent thermal conductivity in the $k_{xy}$ direction can be obtained:

(3) $$Q_{in}=k_{xy}\left\{\frac{T_{hot}-T_{cold}}{P}\right\}\;$$

Here, $T_{hot}$ and $T_{cold}$ are the average temperatures of the left wall and right wall, respectively, and P is the pitch of the TSV cell.

Similarly, the equivalent thermal conductivity in the $k_{z}\;\text{the}\;$ direction of a copper-filled TSV can be calculated. The T is considered as heat flux entering at the top side of the TSV and exiting at the bottom side of the TSV. Using Fourier's law of thermal conduction, the equivalent thermal conductivity in the $k_{z}\;$ direction can be obtained.

(4) $$Q_{in}=k_{z}\left\{\frac{T_{hot}-T_{cold}}{\text{T}}\right\}$$

where $T_{hot}$ and $T_{cold}$ are the average temperature of the top and bottom surfaces, respectively, and T is the thickness of the TSV cell. The thermal conduction along the vertical length of the copper of the TSV/TTSV is isothermal where the conductivity of the copper is uniform. However, the horizontal flow of thermal energy is anisotropic as it hits such barriers as the liner material and silicon substrates.

Zhu *et al.* [16] focused on a unit cell in a power distribution network as an example. They examined the effective thermal conductivity and temperature variations in a 3-D IC stack with different pitches of power delivery or distribution network (PDN) and TSVs. The influence of TCN layers on heat conduction is described in Eqs. (5) and (6).

(5) $$\Delta T=F_{H}.R_{T}$$

(6) $$R_{T}=\frac{l\;}{k\;A}$$

where ΔT, $\;F_{H}$, and $R_{T}$ are temperature differences, heat flow, and thermal resistance, respectively, and l, A, and k are the lengths of the objects, cross-sectional area, and thermal conductivity of copper, respectively.

Zhu *et al.* also reported that, as the number of PDN layers increases, thermal conduction of the interlayer dielectric (ILD)/back end of line (BEOL) in the vertical direction deteriorates. However, because of the change in the cross-sectional areas of the PDN, the thermal conduction in the horizontal direction increases; hence, it can be considered that larger PDN layers enhance the thermal conduction in-plane while reducing vertical thermal conduction. It is evident that the larger PDN layers do not reduce the maximum temperature of the chip or 3-D stacked assembly. However, it is possible to achieve a uniform horizontal temperature. Zhu *et al.* reported that each chip would improve the temperature results by 15% when compared with a case without on-chip PDN. This helped improve the understanding of the problem and provided ideas for resolving such thermal sources. Son *et al.* [17], proposed the novel concept of a thermal transmission line that has no significant impact on the signal integrity of high-bandwidth memory (HBM) TSV channels on-chip. Using this line, it was observed that the thermal integrity improvement for HBM and graphics processing units was reduced by 4.789 °C and 0.057 °C, respectively.

### 3.1 Materials and their size selection

**Figure 1:**
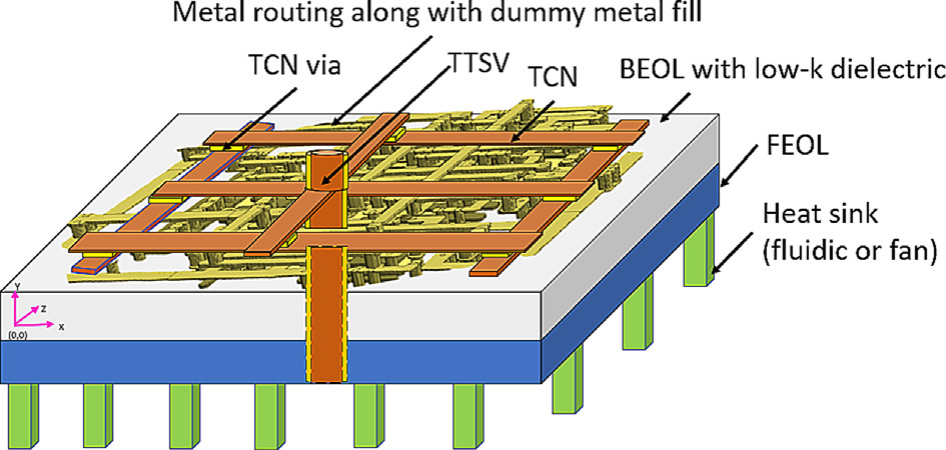
Model of thermal collection network (TCN) for thermal solutions of 2-D IC structures.

A schematic diagram of the model is shown in Figure 1 (not to scale). One pair of TCNs is depicted as being a centrally situated TTSV. The TCNs and TTSV do not leave the BEOL area of the chip. Keeping out of the region of the TTSV could create area overhead on an active chip. The material and sizes chosen for modeling are shown in detail. The heat sink is depicted for demonstration purposes. Depending on the thermal budget of the chip, this could be a fan or a fluidic heat sink.

**Figure 2:**
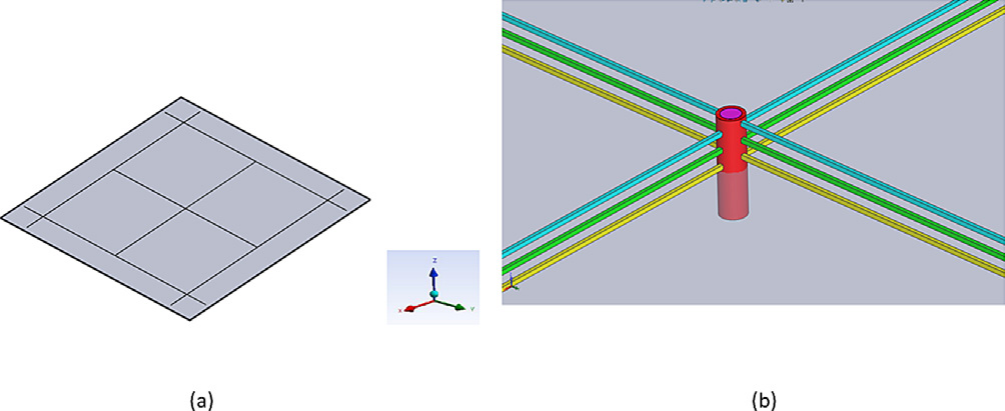
Thermal collection network (TCN) in 2-D IC structure connected to TTSV.

**Figure 3:**
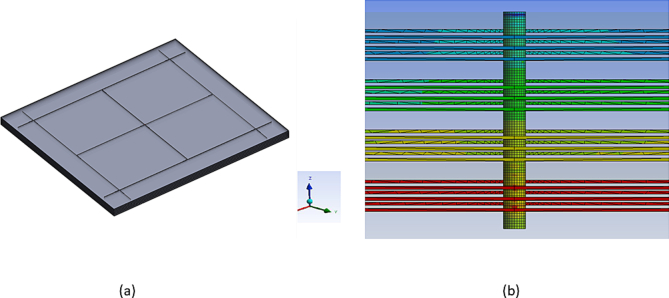
Schematic diagram of four stacked 3-D IC structures connected to TTSV with associated TCNs.

**Figure 4:**
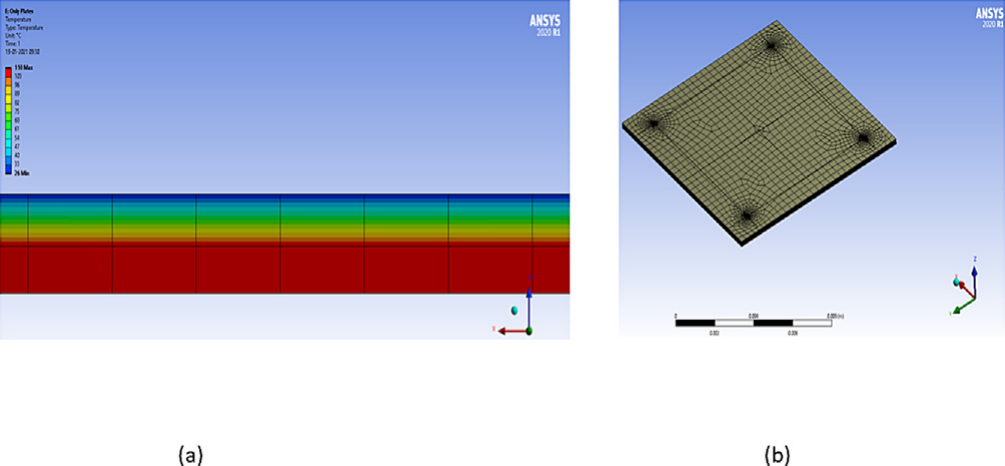
Thermal gradient and mesh grid images in single 2-D IC chip.

Figure 2 shows the structures of the model setup used in the FEM tool. Figure 2(a) is a single chip used in simulations. A topographical view of a three-layer TCN grid connected to a TTSV is shown in Figure 2(b). This also demonstrates how the TTSV should be connected to the TCN layers. Figure 3 shows four stacked 3-D IC structures, and Figure 3(a) indicates the four stacked chips used in the simulations. Figure 3(b) is a cross-sectional view of a three-layer TCN grid connected to a TTSV, in a four-stacked 3-D IC structure (other layers are removed for simplicity). Figure 4(a) shows a temperature image for a single chip. It shows the various thermal gradients inside the 2-D IC chip. The mesh grid for the simulations in the FEM tool is shown in Figure 4(b). This figure reveals that, on the top surface of the 2-D IC chip, four TTSVs are visible in the four corners of the chip.

**Figure 5:**
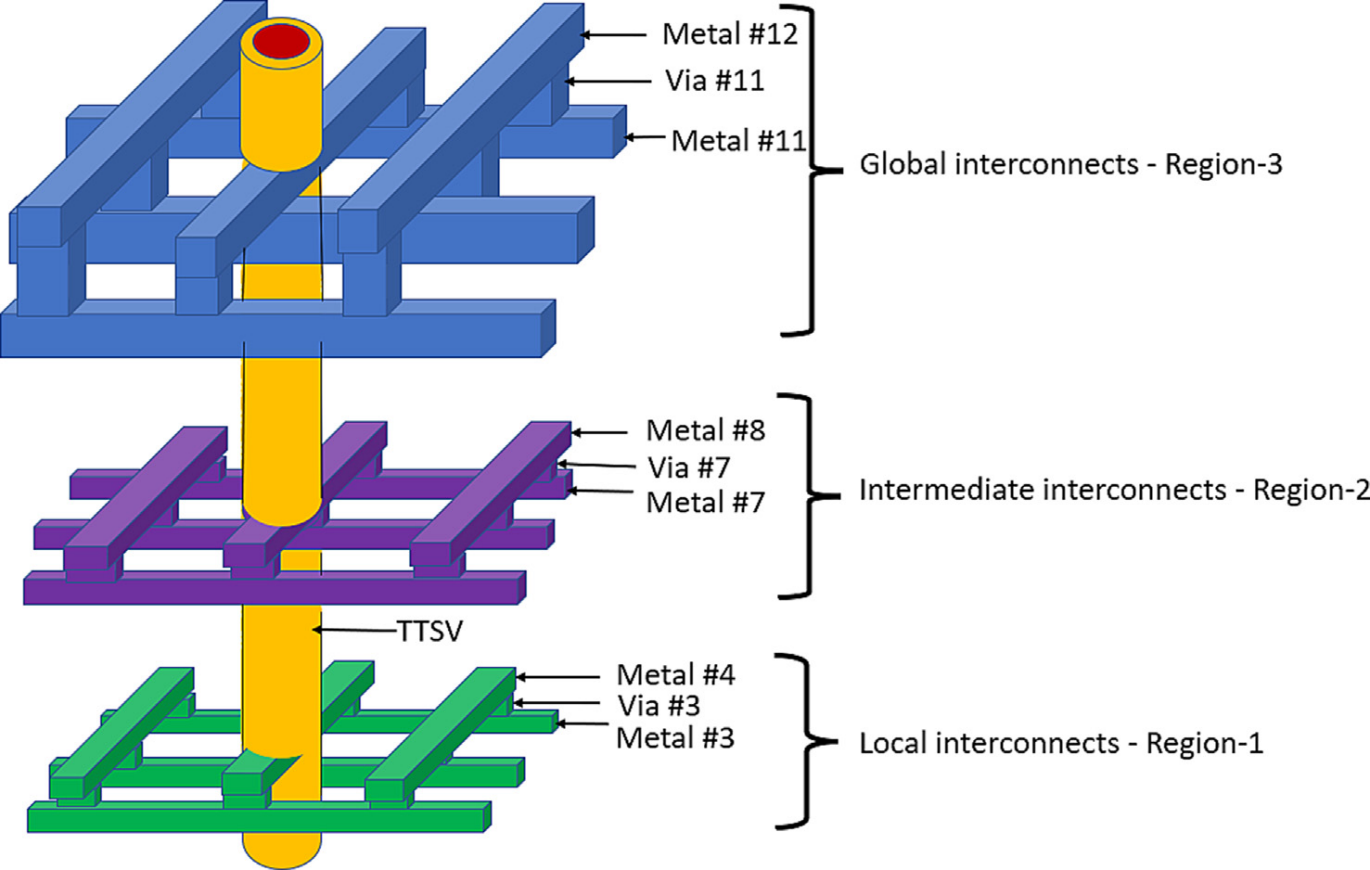
Schematic diagram of TCN rings connected to TTSV in a 2-D IC.

Each of the components in the chip/stack has a sufficient number of mesh points. In coarse meshing, at least 25,000 mesh points are present in each chip of the stack while using the FEM tool. The number of metal layers is increased to as high as 16–20 layers in ≤7-nm deep submicron chip design technology. Because of the increased resistance of metal layers with narrow widths, more metal density is needed in the PDN. Accordingly, in the modeling configuration, the BEOL or ILD thickness was set to 10 μm. This is because vias are individually short in height, as illustrated in Figure 5, especially in local and intermediate interconnects. These vias are taller in the case of global interconnects because of the greater pitch of global interconnects. The height of the vias is usually the same as the width of the top metal of the pair. Considering all fin field-effect transistors, the base layer devices have a 20-μm thickness in the substrate area. Low-k dielectrics are commonly used for BEOL/ILD layers. For ease of use, a low-k dielectric, such as a carbon-doped oxide with k = 0.39 W/m.K was used here. Mismatches in CTE would be an issue because copper is being used in the TCN within the BEOL/ILD layer. However, it would not cause much addition from the TCN metal pairs because there are other routed metal pairs inside the BEOL. Additional TTSV formation copper, however, is an added metal overhead inside the BEOL section of the chip. Experiments are conducted on very thin chips, with various design thicknesses, as referred to in a literature survey [10]. In some foundries, a thinner base layer might still be used. An experiment was carried out in this study using four such chips stacked in a “face top” pattern. The chips are connected with a vertical TTSV of diameter 10 μm with a liner material of 2 μm (1 μm on each side) and 8 μm of copper filler in a monolithic IC structure, with a total height of 30 μm, for a single chip. The practical dimensions have been considered for the TTSVs as well as the chips, and the footprint area $A_{0}$ is considered a large system on a chip (SoC). The chip area is 10 mm by 10 mm, with a thickness of 30 μm, for the analysis and discussion. For the concept of these 3-D IC structures, it has been considered that heat sink and fan assemblies should empty the heat transferred to them efficiently. The heat sink could be of the encapsulated/liquid cooling type. This type is ideal for pre-empting thermal energy, and, as a result, the entire process works flawlessly.

These conditions are presumed in all the thermal paths of the various experiments in this study. The operating voltage for each stacked chip is 0.765 V. Each chip has four setups and five hold timing corners, according to the six-sigma multicorner-multimode, with a target frequency of 1.2 GHz.

### 3.2 Reasons to position three TCNs

TCN rings with horizontal and vertical metal shapes are attached to the TTSV in a single chip/die, as shown schematically in Figure 5 (not to scale). To position the TCN metal inside a chip, it is necessary to choose three regions within the BEOL/ILD area, as shown in Figure 5. The reasons for selecting specific metal layers are discussed in detail later. For simplicity, a pair of metals (for example, Metal #3 and Metal #4) is considered. These are local interconnects. Metal#1 and Metal #2 would be dedicated to the power grid to supply power/ground to standard cells of the chip. SRAMS typically has blockages until Metal #4. To capture physical memory activity heat, as well as standard cell activity heat, Metal #3 was chosen, and Metal #4 for Region 1, as shown in Figure 5. The reason for choosing Metal #7 and Metal #8 is that they are middle/intermediate level interconnects in Region 2 as shown in Figure 5. The reason for choosing Metal #11 and Metal #12 is that they are higher/global-level interconnects in Region 3, as shown in Figure 5, to capture any thermal energy generated by some of the other devices, such as ternary content-addressable memories and other IPs. Usually, these IPs have blockages until Metal #10 or Metal #11. Furthermore, Metal #12 and above are dedicated to the power grid, and the metals have fewer empty tracks. Within the chip, there can be many partitions and subblocks. The width of such a metal stripe for a block/partition/chip can be calculated using Eqs. (1) and (2).

For simplicity, the widths of TCNs have been considered in a way explained further. Looking from the top level of the chip, Metal #11 and Metal #12 have widths of 4 μm. Metal #7 and Metal #8 have 2-μm widths. Metal #3 and Metal #4 have widths of 1 μm. The pitches and widths of the aforementioned metals are even smaller in industry-standard foundries, particularly in the case of 7 or 5 nm. These widths and pitches may or may not correspond to the standard electrical widths and pitches for certain metal pairs. They can be made bigger if necessary, and this means double/quadruple widths of pitches unless they are supported by the design rule checks of the technology node. Another technique to reduce thermal resistance is to use a large number of TCN pairings in a single metal layer pair. The TCN layer should not be considered as a reservoir that absorbs thermal energy. Furthermore, because the TCNs are connected to the TTSVs, which continuously pre-empt the generated heat from the sources in the thermal circuits, self-heating is not a major concern. Vias, which link the top and bottom metals, are typically approximately of the same height and width. This is because, in general chip design protocols, metal widths can be increased. Therefore, the height of the vias was kept the same as the width of the metal pairs in this study. Pitches of metal pairs in the plane were put at a distance of approximately 200 μm from the edges of the chip. When stacking four chips, a gap of 20 μm was formed between two chips, which were filled with polymer. Electrical TSV/TSV (ETSV/TSV) was considered to connect through chips using microbumps in heterogeneous 3-D IC structures. Dummy microbumps with an air gap or polymers for providing mechanical support could be present in such an environment.

**Figure 6:**
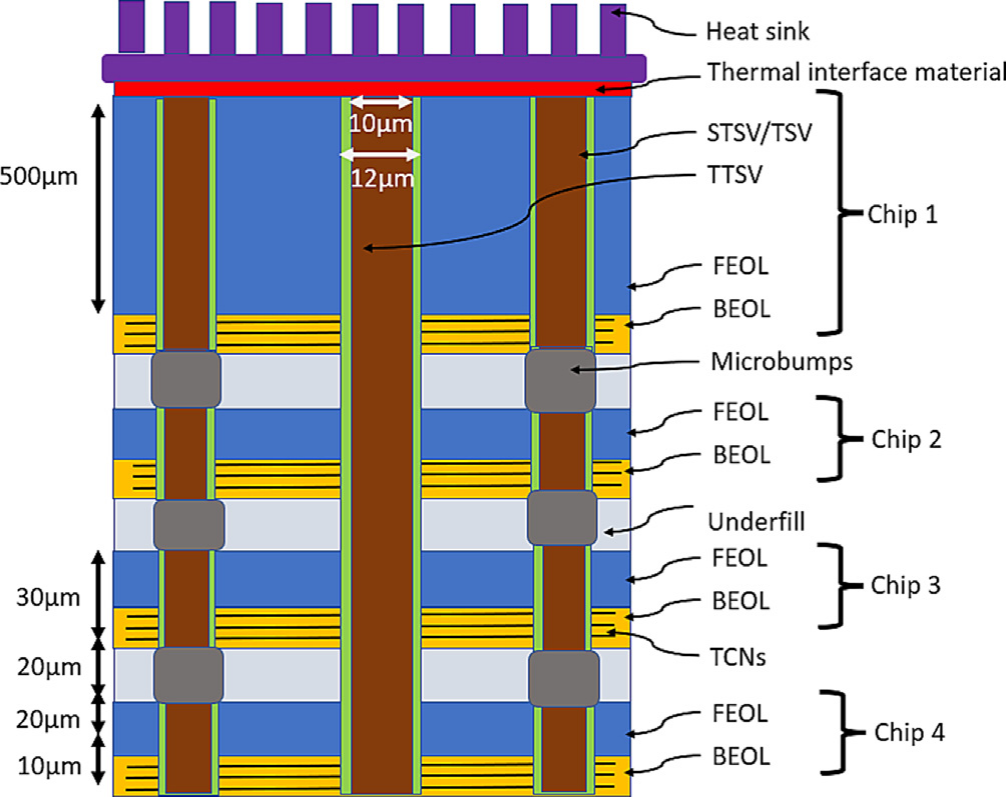
Thermal collection network in a monolithic 3-D IC with a stack of four chips.

Figure 6 depicts four stacked 3-D ICs, from Chip 1 to Chip 4, and the dimensions taken into consideration for the various components in the schematic diagram (not to scale). A polymer material with a thermal conductivity of 0.5 W/m.K was used for underfilling.

### 3.3 Applied boundary conditions

**Figure 7:**
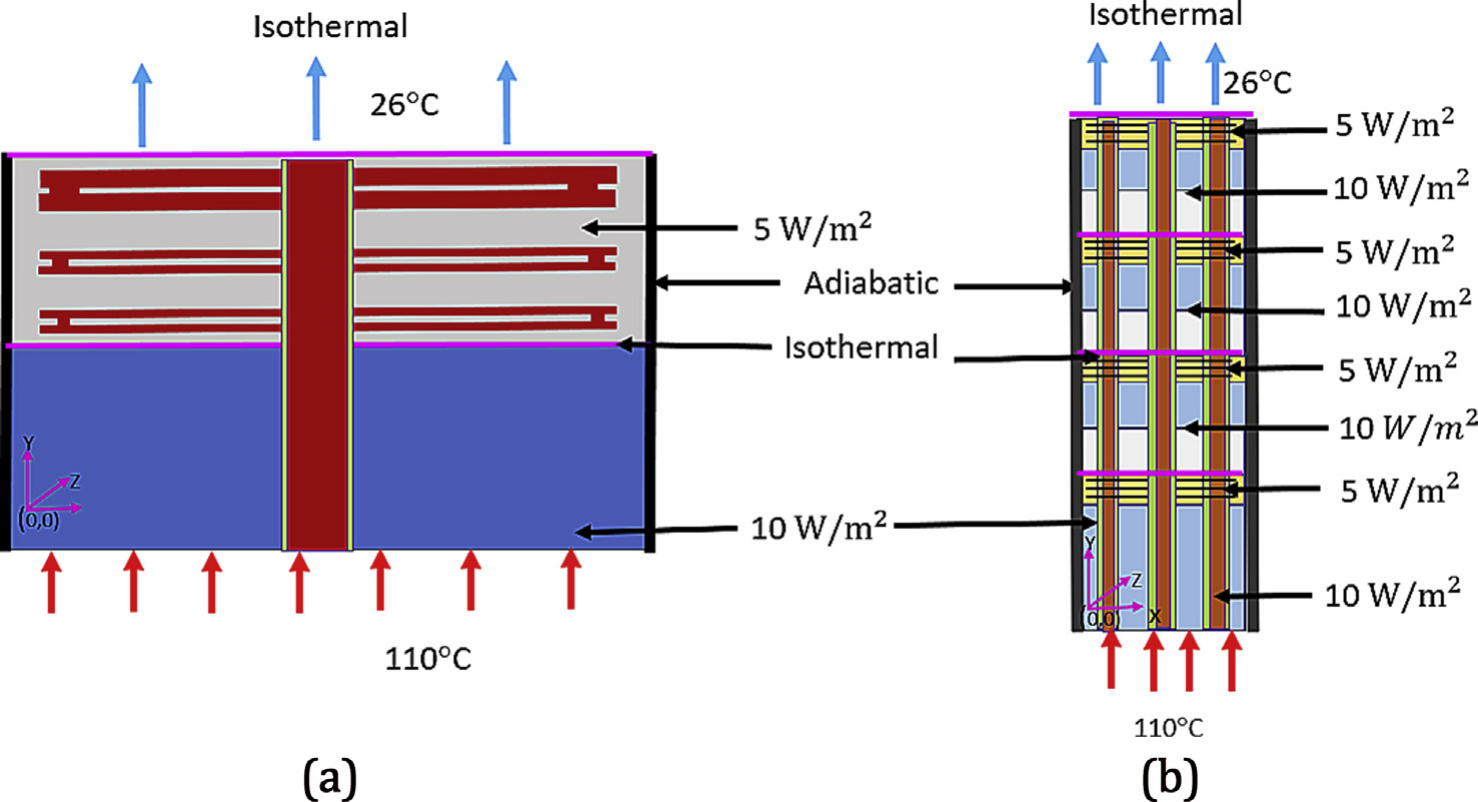
Boundary conditions applied to the 2-D IC chip and 3-D IC stacked with four chips.

On top of each chip/stack, a heat flux of 5 $\text{ ​W}/{\text{m}}^{2}$ was applied as a boundary condition to achieve metal/power grid heating. The bottom chip/stack is subjected to 10-$\text{W}/{\text{m}}^{2}$ heat flux as a result of base layer activity. Physically, the heat sink could be located on top of a silicon chip, coupled with the fan to cool the heat sink. This arrangement can be imagined as an air/liquid coolant flowing to the bottom of the chip/stack. In terms of physical architecture, this means the 2-D/2.5-D/3-D IC would have an air/liquid cooling heat sink on top and natural convection cooling on the bottom. The bottom layer of the four-chip stack has a maximum temperature of approximately 110 °C applied because of the flip-chip packaging, while the top layer has a minimum temperature of 26 °C. Furthermore, the ambient temperature is maintained at 25 °C. All four sidewalls of the stack are in adiabatic thermal isolation, and heat flux is uniform and normal to the surfaces. Interlayers are isometric by nature, assuming that the base layer devices and interconnects produce a uniform heat flux. Joule heating appears in each chip and throughout the entire stack. The device power density is applied to the top of each Si layer, whereas the interconnect heat flux is applied to the BEOL/ILD layer. Figure 7(a) illustrates (not to scale) the boundary conditions for a monolithic 2-D IC chip. Figure 7(b) illustrates the applied boundary conditions in a 3-D IC structure in a visual form. The material sizes and conductivities used in the FEM analysis are summarized in Table 1. Throughout this model study, the methodology followed was to vary the number of TCN grids from zero to three. These are distributed across three vertical sections of the chip. The number of TTSVs connecting to them is varied from zero to five, and they are connected with various TCN grid patterns.

**Table 1:** Summary of properties and dimensions of materials.

Parameter	Material	Size/Conductivity
TTSV diameter	Copper	10 μm
Filler thickness	Copper	8 μm
Liner thickness	${\text{SiO}}_{2}$	1 μm
Die dimensions	Silicon	10 mm∗10 mm
Substrate height	Silicon	20 μm
Underfill height	Polymer	20 μm
ILD Layer (CDO)	Carbon Doped Oxide	10 μm
**TTSV filler conductivity**	Copper	400 W/m.K
**TTSV liner conductivity**	${\text{SiO}}_{2}$	1.4 W/m.K
**ILD layer conductivity**	Carbon Doped Oxide	0.39 W/m.K
**Silicon Conductivity**	Si	130 W/m.K
**Silicon dioxide Conductivity**	${\text{SiO}}_{2}$	1.4 W/m.K
**Underfill Conductivity**	Polymer	0.5 W/m.K

## 4 Results, analysis, and discussion

The thermal performance of various TCN rings along with TTSV stacked systems were studied by FEM simulations. Graphs and data are analyzed in the following sections.

### 4.1 Thermal performances of TCNs in 2-D/2.5-D ICs

**Figure 8:**
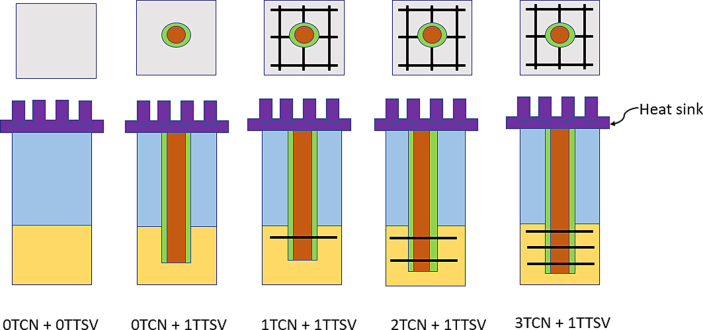
Schematic diagram of the 2-D IC/2.5-D ICs with multiple TCNs and a single TTSV.

The thermal performance of TCNs can be studied on 2-D/2.5-D IC structures, as shown in Figure 8. The power dissipation in the substrate/base layer of the active region of IC heat flux is 10 $\text{ ​W}/{\text{m}}^{2}$, and it is applied as a major heat source. On-chip PDN is a minor heat generator. The heat flux of power dissipation applied is 5 $\;\text{W}/{\text{m}}^{2}$ for performance checking. The boundary conditions are shown in Figure 7(a). Figure 8 depicts TCNs with dimensions specified as in the computational setup in a cross-sectional view. These are located in three places in the 2-D IC. There are five different forms of studies with names ranging from “0TCN + 0TTSV” to “3TCN + 1TTSV.” Uniform thermal generation was used in the first stage of this model study. Designers may render more dedicated or localized TCNs in physical-world chip designs to create more thermal runways around established peak power consumption areas or hot spots. IR drop analysis software can easily define and evaluate such local hot spots. A greater number of TCNs were added in the vertical direction, along with more metal staples, to maximize heat transfer. The larger the TCNs, the more uniform the heat in the horizontal direction.

**Figure 9:**
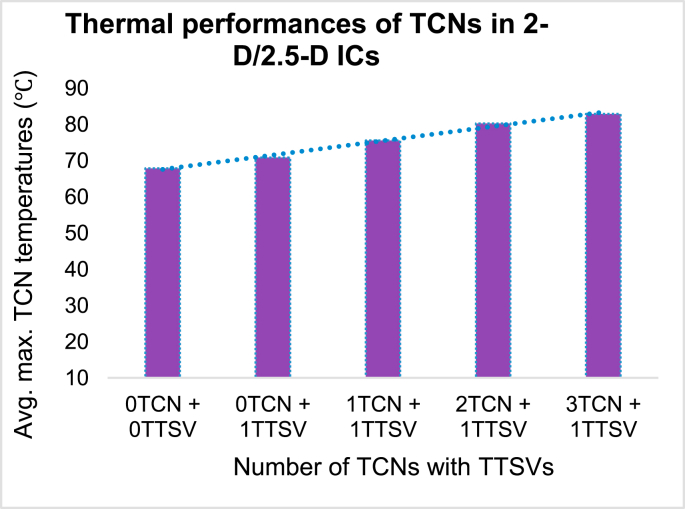
Thermal performances of 2-D/2.5-D ICs with multiple TCNs and a single TTSV.

When there is no TCN and no TTSV in a single chip, the average maximum temperature stored within the ILD/BEOL region is as high as 67.88 °C, as shown in Figure 9. That means that, with 110 °C applied at the base, the majority of the temperature is released inside the substrate. The ILD temperature suction increases by 71.27 °C when a single TTSV is introduced. When TCNs are added to the lower layers of the chip, the ILD overall average temperature rises to 75.33 °C. As TCN rings are inserted in two more locations, such as the chip's intermediate and top layers, the maximum temperature suction reaches 82.91 °C. It is clear that, if dedicated thermal runways are used, they will absorb more thermal energy. As a result, doing so could be the solution to thermal mitigation in 2-D/2.5-D ICs. In later stages, the heat must be sucked into the heat sink and fan assembly.

### 4.2 Thermal performances of multiple TTSVs and multiple TCNs in 2-D/2.5-D ICs

**Figure 10:**
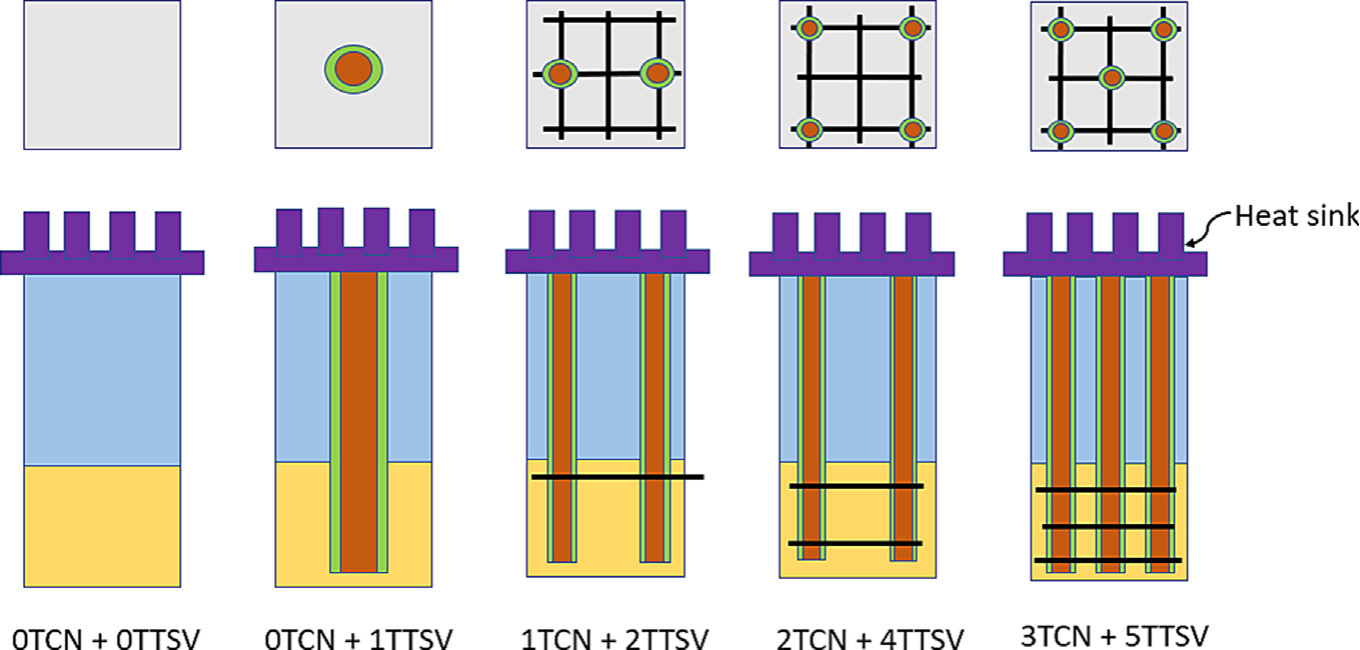
Schematic diagram of the 2-D/2.5-D ICs with multiple TCNs and multiple TTSVs.

Figure 10 shows a cross-sectional diagram of a 2-D IC with multiple TTSVs and multiple TCN rings. There are five different types of study identified from “0TCN + 0TTSV” to “3TCN + 5TTSV”. When there is no TTSV, the introduction of a single TTSV, followed by two, four, and five TTSVs, along with three rings of TCNs, could cause disruptive changes. When multiple TTSVs are used, the empirical Eqs. (7) and (8) reflect the equivalent thermal conductivities inside the chip [3].

(7) $$k_{eqv,Z\;\left(chip\right)}=150+180\;\left(D^{-2}P^{-2}\right)$$

(8) $$k_{eqv,\;X\left(chip\right)}=k_{eqv,\;Y\left(chip\right)}=150+105\;\left(D^{-2}P^{-2}\right)$$

where P is the pitch of the TTSVs, and D *=* $\;\left(D_{1}+D_{2}\right)/2$ is the annotation of the diameters of the top ($D_{1})\;$ and bottom ($D_{2})$ of the TTSV, while considering the practical tapered width of the TTSVs.

**Figure 11:**
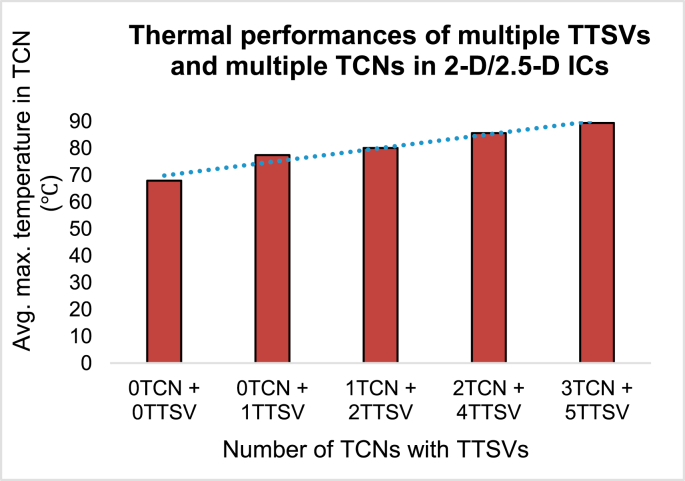
Thermal performances of the 2-D/2.5-D ICs with multiple TTSVs and multiple TCNs.

The equations reveal that, when multiple copper-filled TTSVs are used, the thermal conductivity of the chip in the Z-direction (vertical) is much faster than the thermal conductivity of the chip in the X- and Y-directions (horizontal). Figure 11 shows that the ILD maximum average temperature of the chip was 67.88 °C when there was no TTSV and a TCN ring grid. By introducing one, two, four, and five TTSVs and increasing the TCN grids to three, the average ILD temperature of each 2-D IC drastically increases to 89.34 °C, as shown in Figure 11. Thereafter, this heat can be transferred to the heat sink and fan assembly in the following stages.

This is the reason for having more TTSVs in 2.5-D structures. An example of such an IC is a network routing chip that can range from 50 0 to 900${\text{ ​mm}}^{2}$ in size.

These chips might be integrated with HBM stacks in a 2.5-D IC configuration; hence, thinly sliced chips with wider widths require more TTSVs. This postulate is compatible with HBM in a 2.5-D package because of the thermal compression bonding using nonconductive films that could accommodate the proposed model.

### 4.3 Thermal performances of TCNs in 3-D IC systems

**Figure 12:**
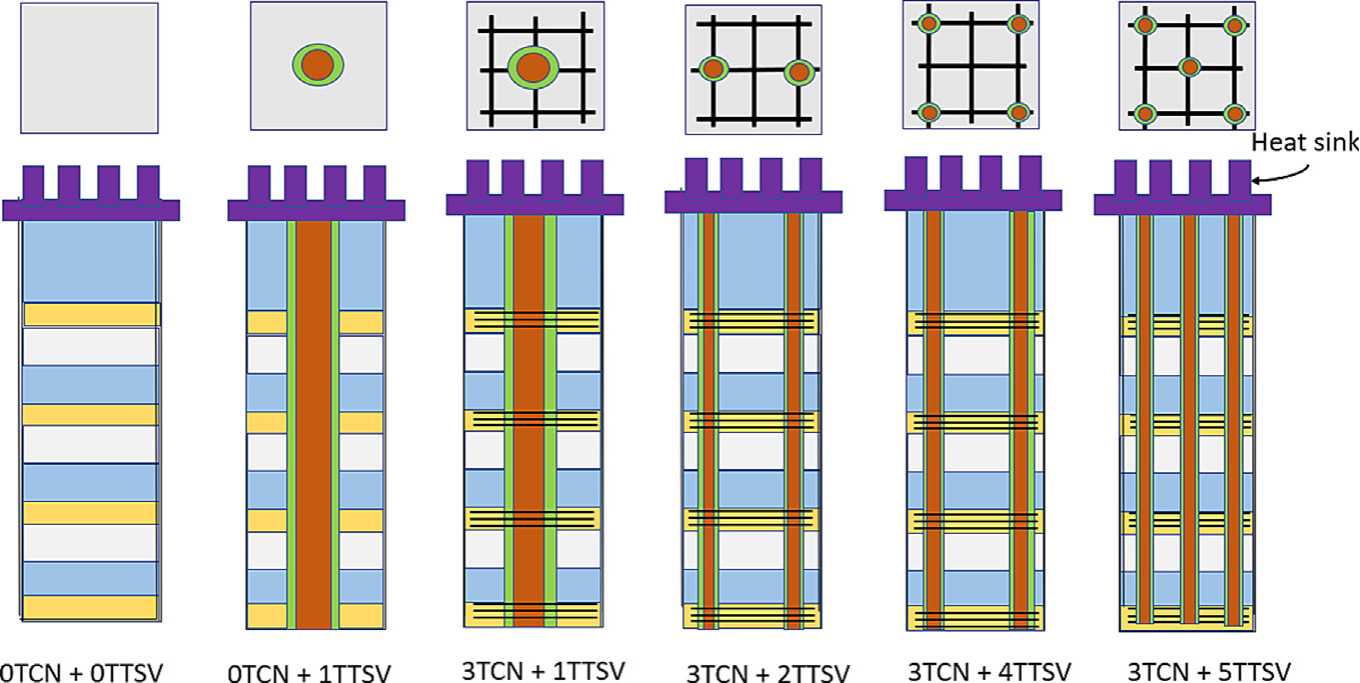
Schematic diagram of the 3-D IC structures with multiple TTSVs and three rings of TCNs.

After the brief research on the 2-D or 2.5-D IC structure, we turned our focus on 3-D IC structures because thermal mitigation is crucial. In Figure 12, it is shown that Chip 1 to Chip 4 has been bonded in a pattern where the “face top” is shown in the cross-sectional view. The simulations in Figure 12 assumed the presence of signal TSVs inside the 3-D IC. The figure does not show such signal TSVs to emphasize the importance of the TTSV and TCN structure. The experiments are listed as “0TCN + 0TTSV” to “3TCN + 5TTSV.” For clarity, four of the stacked chips were connected in such a way that a 3-D IC structure could be formed with the help of one single TTSV or with five TTSVs. This could be extended to 3-D IC structures with a monolithic or heterogeneous formation. It is also possible to link the TTSV with microbumps when forming a single vertical rod. However, in this case, microbump materials are considered because they have a negligible impact on the thermal mitigation connecting TTSVs/TSV, as in earlier reports [15, 16].

**Figure 13:**
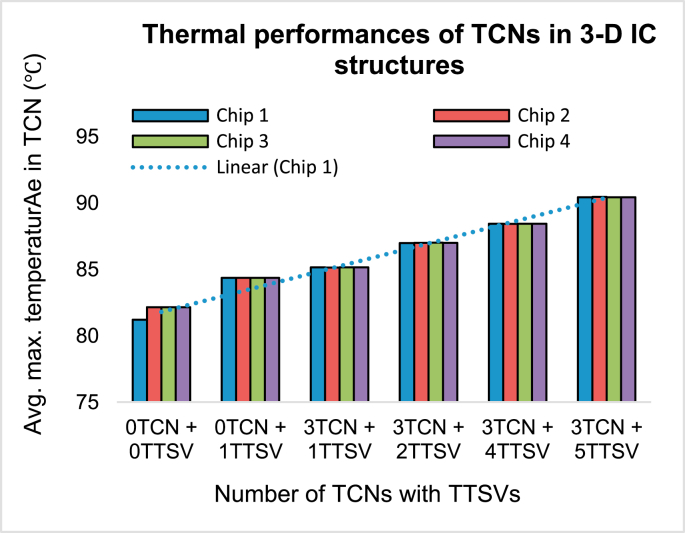
Thermal performances of 3-D IC structures with multiple TTSVs and three rings of TCNs.

Figure 13 shows the average maximum temperatures inside the TCNs of a single TTSV connected with three rings of TCNs to five TTSVs connected with three rings of TCNs inside the four chip stacked structure. The average temperature rose in the TCNs to 91.43 °C. Without these structures, the average temperature remained 81.20 °C. As previously mentioned, this heat is eventually transferred to the heat sink. Thus, without the capillary fluidic solutions inside 3-D IC stacks, there could not be a complete and novel solution to the thermal mitigation problem of 3-D ICs.

### 4.4 Junction temperatures of TTSVs in 3-D IC systems

The junction temperature is an important factor because multiple TSVs and TTSVs are used in the 3-D stacks. The annotation of junction temperature can be described as

(9) $$R_{therm}=\frac{T_{junct}-T_{ambi}}{H_{flow}}\;$$

Where ${\text{R}}_{therm}\;$ is the thermal resistance of the stack, $H_{flow}$ is the generated heat of the stack, and $T_{junct}$ and $T_{ambi}$ are the stack junction and ambient temperature, respectively.

**Figure 14:**
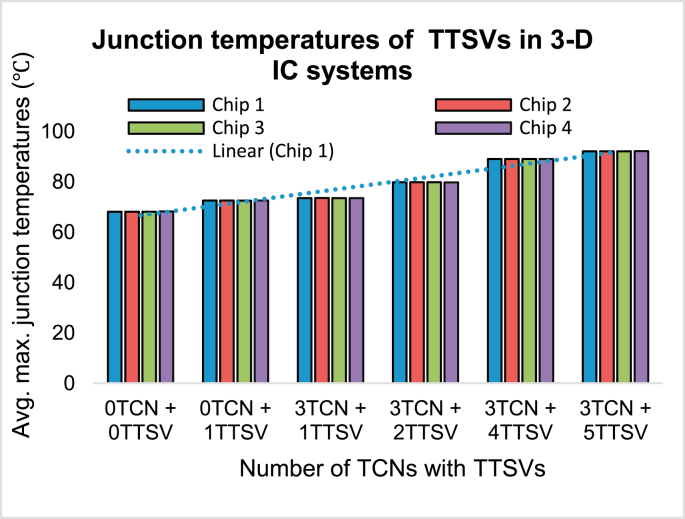
Junction temperatures for four stacked TTSV chips (uniform heat sources).

The journey from the “0TCN + 0TTSV” to the “3TCN + 5TSV” experiment is shown in Figure 14. It has been proved that the temperature distribution for various TTSV in the stack fashion is always uniform. It has also been noticed that, when the 3-D stack is examined without the presence of any TTSVs or TCNs, the average maximum junction temperature goes up to 68.04 °C; however, within the TTSVs, the maximum average junction temperature goes up to 92.09 °C when properly introduced to all five TTSVs.

A TTSV has continuous uniformity in the vertical direction of the heat flow for the aforementioned reasons. In the case of TSVs, a junction always appears shortly after durability ends. The signal must pass through different media, and that causes the junction temperature to hold the most significant role. The junction temperature process requires a huge budget, which results in the integration of only few 3-D IC stacks as others chips get eliminated. However, the suction is the path preceding the next step of the heat distribution. If it is pre-empted effectively, then more stacks could be compounded than the current limitation based on the junction temperature. Table 2 summarizes all the experiments done. The advantages of the thermal runways are documented for thermal runways without TCNs and TTSVs.

**Table 2:** Model results in brief.

Experiment names	Without TCN/TTSV	With TCN/TTSV	Benefits compared to without TCN and TTSV (%)
	Average maximum temperatures (°C)		
Thermal performances of TCNs in 2-D/2.5-D ICs	67.88	82.91	22.13%
Thermal performances of multiple TTSVs and multiple TCNs in 2-D/2.5-D ICs	67.88	89.34	31.62%
Thermal performances of TCNs in 3-D ICs	81.20	91.43	11.36%
Junction temperatures of TTSVs in 3-D ICs	68.04	92.09	35.34%

## 5 Conclusions

For the design of a thermal grid associated with a collective network, a model has been proposed along with the necessary information about physical attributes that can be subsequently used in designing optimal thermal collection/reception networks for an “n” metal layer system. This includes such design constraints as power, the budget of the IR drop, and supply voltages for different types of SoCs, system-in-a-package packages, heterogeneous/nonmonolithic structures, and monolithic 2-D/2.5-D/3-D IC structures.

FEM analysis resulted simultaneously in 22.13% more performance in thermal energy suction for three rings/layers of TCNs. If five TTSVs are incorporated with three rings/layers in the TCN grid, then the thermal suction reaches a percentage of 31.62% for the 2.5-D monolithic ICs. When TCNs are introduced in the monolithic 3-D IC system, thermal energy suction increases to 11.36% of the total system package and 35.34% of the heat suction in junctions of TTSVs (as detailed in Table 2).

In the physical design of the chip, the novel innovative TCN must be established just after the PDN associated with TTSV connectivity. After these steps, routing tasks can be completed for the remaining places. However, the foundry procedure is beyond the scope of this study. In future work, TCNs with additional diagonal network connections should be added in the TTSV structure for better thermal heat management compliant with Manhattan routing. The use of analytical models instead of an FEM model could also be within the scope of this project.

The limitation of the current methodology includes the possibility of a wide area overheating because of the inclusion of additional metal stripes formed because of the TCN, as well as TTSV formation. TCNs do not need to be considered as overhead because of the Manhattan routing of the BEOL layers, which include unused metal pitches and can create a crowded situation with metals for supporting design for manufacturability/yield approaches. Therefore, TCNs can be categorized as BEOL layers, and very few metal tracks can be used in them. However, the TTSV formation along with the keep-out region in the active construction of the chip could be considered an area overhead. Another limitation is the uncertainty of the performance. If the subsequent stages of the fan–heat–sink assemblies fail, then the thermal power transmitted mechanically using this particular process could result in high thermo-mechanical stresses inside the chip. To avoid such situations, an extremely efficient heat sink encapsulated with cooling material could be used.

The purpose of this study was to invent specific TCN rings/grids and thermal runway paths for the thermal absorptions because these help improve the temperature distribution in IC systems. In the research process, thermal energy is pre-empted swiftly and efficiently. Thus, alleviation of thermal problems and thermal management of 2-D/2.5-D and 3-D IC structures can be done effectively.

## Declarations

### Author contribution statement

Chandrashekhar V. Patil: Conceived and designed the experiments; Performed the experiments; Analyzed and interpreted the data; Wrote the paper.

Suma M. S: Analyzed and interpreted the data; Wrote the paper.

### Funding statement

This research did not receive any specific grant from funding agencies in the public, commercial, or not-for-profit sectors.

### Data availability statement

Data associated with this study has been deposited at Mendeley under the accession URL https://data.mendeley.com/datasets/tjfrts9pvy/3.

### Declaration of interests statement

The authors declare no conflict of interest.

### Additional information

No additional information is available for this paper.
